# Complete Genome Sequence of Salmonella enterica Siphophage Shemara

**DOI:** 10.1128/MRA.01518-19

**Published:** 2020-02-06

**Authors:** Michael Chung, Yicheng Xie, Heather Newkirk, Mei Liu, Jason J. Gill, Jolene Ramsey

**Affiliations:** a Center for Phage Technology, Texas A&M University, College Station, Texas, USA; Queens College

## Abstract

Here, we present the annotated genome of Shemara, a siphophage of Salmonella enterica. The Shemara genome is 44 kb with 83 predicted protein-coding genes. At the nucleotide and amino acid levels, Shemara is most similar to phages in the *Guernseyvirinae* subfamily.

## ANNOUNCEMENT

Here, we describe the genome of phage Shemara, a siphophage that infects the Gram-negative bacterium Salmonella enterica. Humans are typically exposed to S. enterica, one of the most common causes of foodborne illnesses, through infected livestock or plant food products (1). Significant progress has been made in the investigation and application of phages as natural biocontrol agents in food products to reduce the incidence of disease (2).

Bacteriophage Shemara was sourced from a south Texas beef cattle feedlot. Shemara was isolated against S. enterica serotype Anatum, which is associated with recent human disease outbreaks (3, 4). Host bacteria were cultured on tryptic soy broth or agar (Difco) at 37°C with aeration, and phage isolation and propagation were done using the soft-agar overlay method (5). The phage morphology was viewed after negative staining with 2% (wt/vol) uranyl acetate under transmission electron microscopy at the Texas A&M Microscopy and Imaging Center (6). Genomic DNA was purified by the shotgun assembly modification of the Wizard DNA kit (Promega) described by Summer (7). After purification and library preparation using an Illumina TruSeq Nano low-throughput kit, the phage DNA was sequenced on the Illumina MiSeq platform using paired-end 250-bp reads. The 128,596 total resulting sequence reads were quality controlled with FastQC (www.bioinformatics.babraham.ac.uk/projects/fastqc) and trimmed with the FASTX Toolkit v0.0.14 (http://hannonlab.cshl.edu/fastx_toolkit/). A single contig was assembled with SPAdes v3.5.0 to 130-fold coverage at default parameters, including k-mers of 21, 33, and 55 (8). A second contig, assembled using k-mers of 99, 103, and 127, with 36.7-fold coverage, was opened in a different place, allowing complete closure of the genome sequence. Despite this, PhageTerm analysis with raw sequencing reads was unable to assign the terminus type (9). Identification of gene sequences was done with MetaGeneAnnotator v1.0, GLIMMER v3.0, and ARAGORN v2.36 (10–12). TransTermHP v2.09 was used to predict the presence of rho-independent terminators (13). Gene function was predicted using conserved domain searches with InterProScan v5.22-61 and BLAST similarity searches with a 0.001 maximum expectation value threshold against the NCBI nonredundant and UniProtKB Swiss-Prot and TrEMBL databases (14–16). TMHMM v2.0 predicted transmembrane domains (17). Annotation tools were used in the Galaxy and Web Apollo instances hosted by the Center for Phage Technology (https://cpt.tamu.edu/galaxy-pub/) (18, 19) with default parameters, unless otherwise stated. ProgressiveMauve v2.4.0 assessed the whole-genome nucleotide similarity (20).

Shemara is a siphophage, and its genomic contig has a length of 44,342 bp with 51.4% G+C content. With 83 predicted gene-coding sequences, 34 of which were assigned a putative function, its coding density is 95.8%. Shemara is most similar to several phages within the *Guernseyvirinae* subfamily (21), namely, *Salmonella* virus VSiP (GenBank accession number MH424444), with 72% nucleotide identity and 59 shared proteins, and *Salmonella* phage St162 (GenBank accession number MF158037), with 72.1% nucleotide identity and 53 shared proteins.

### Data availability.

The genome sequence and associated data for phage Shemara were deposited under GenBank accession number MN070121, BioProject accession number PRJNA222858, SRA accession number SRR8869235, and BioSample accession number SAMN11360359.
